# CycPeptMPDB: A Comprehensive Database of Membrane Permeability of Cyclic Peptides

**DOI:** 10.1021/acs.jcim.2c01573

**Published:** 2023-03-17

**Authors:** Jianan Li, Keisuke Yanagisawa, Masatake Sugita, Takuya Fujie, Masahito Ohue, Yutaka Akiyama

**Affiliations:** † Department of Computer Science, School of Computing, Tokyo Institute of Technology, Meguro-ku, Tokyo 152-8550, Japan; ‡ Middle-Molecule IT-based Drug Discovery Laboratory (MIDL), Tokyo Institute of Technology, Meguro-ku, Tokyo 152-8550, Japan

## Abstract

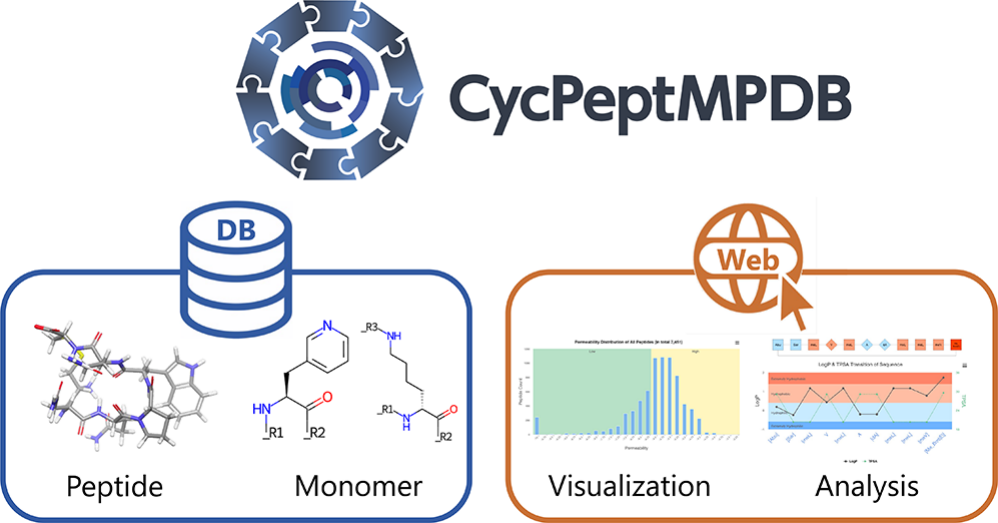

Recently, cyclic peptides have been considered breakthrough drugs because they can interact with “undruggable” targets such as intracellular protein–protein interactions. Membrane permeability is an essential indicator of oral bioavailability and intracellular targeting, and the development of membrane-permeable peptides is a bottleneck in cyclic peptide drug discovery. Although many experimental data on membrane permeability of cyclic peptides have been reported, a comprehensive database is not yet available. A comprehensive membrane permeability database is essential for developing computational methods for cyclic peptide drug design. In this study, we constructed CycPeptMPDB, the first web-accessible database of cyclic peptide membrane permeability. We collected information on a total of 7334 cyclic peptides, including the structure and experimentally measured membrane permeability, from 45 published papers and 2 patents from pharmaceutical companies. To unambiguously represent cyclic peptides larger than small molecules, we used the hierarchical editing language for macromolecules notation to generate a uniform sequence representation of peptides. In addition to data storage, CycPeptMPDB provides several supporting functions such as online data visualization, data analysis, and downloading. CycPeptMPDB is expected to be a valuable platform to support membrane permeability research on cyclic peptides. CycPeptMPDB can be freely accessed at http://cycpeptmpdb.com.

## Introduction

The average costs associated with developing a new drug from discovery to market far exceed one billion dollars, and the process can take more than 12 years.^1^ One of the difficulties is the decreasing number of targets for conventional small-molecule and antibody drugs. It has been reported that approximately 80% of all disease-relevant human proteins, including those involved in intracellular protein–protein interactions (PPIs), cannot be tackled by conventional small-molecule drugs and antibody drugs.^2^ Cyclic peptides have recently emerged as a promising pharmaceutical modality because they can access the targets which have traditionally been considered “undruggable.” Cyclic peptides exhibit several pharmacological characteristics that distinguish them from other classes of therapeutic molecules.^3,4^ For example, cyclic peptide drugs have a broader interaction surface than small-molecule drugs and thus may function as inhibitors with high affinity and selectivity for PPIs. Moreover, these drugs can be produced relatively inexpensively compared to antibody drugs due to their synthetic accessibility. Previously, cyclic peptide drugs were primarily natural peptides or mimics of those peptides.^5^ The random nonstandard peptides integrated discovery (RaPID) system has made it possible to synthesize a large number of cyclic peptides from an extremely diverse amino acid library and quickly select strong binders against arbitrarily chosen therapeutic targets.^6^ Using this approach, numerous research teams and pharmaceutical companies are actively engaged in cyclic peptide drug discovery. Currently, at least one cyclic peptide is approved for clinical use every year on average.^7^

However, the cell membrane permeabilities of common cyclic peptides are lower than the desired range for drugs, which severely limits their biological applicability.^8^ Cell membrane permeability is one of the most important indicators for assessing oral bioavailability and the possibility of intracellular targeting. In general, relatively large cyclic peptides exceeding 10 amino acid residues have high target affinity; however, they tend to have low membrane permeability due to their large size. Although cyclic peptides have a variety of membrane permeation mechanisms, passive diffusion driven by differences in concentration is one of the most prevalent mechanisms.^9^ Even for passive diffusion, the process by which cyclic peptides permeate membranes is not well understood. In contrast to linear peptides, many cyclic peptides have an environment-dependent property called the “chameleonic” property, namely, the ability to change their molecular conformation and hydrophobicity in response to the surrounding environment. These chameleonic cyclic peptides prefer an “open” conformation in aqueous environments, where the polar groups are exposed to the outside and interact with water molecules to enhance water solubility, and a “closed” conformation in hydrophobic environments, where the polar groups are shielded with intramolecular hydrogen bonds or lipophilic side chains, leading to improved membrane permeability.^10−12^ Therefore, the most common strategy to increase membrane permeability has been to allow cyclic peptides to have a “closed” structure inside the cell membrane by shielding the exposed NH group with backbone *N*-methylation.^13,14^ In addition, some strategies have been tested to improve membrane permeability by amide-to-ester substitution^8^ or changing the structure of the side chain to form an intramolecular hydrogen bond with the main chain.^15^

In the early stages of drug development, it is important to select candidate compounds with high membrane permeability; however, it is a costly process to randomly measure large numbers of peptides using biochemical assays such as the parallel artificial membrane permeability assay (PAMPA). Hence, a rapid computational method for predicting membrane permeability is required. Computational approaches for predicting the permeability of cyclic peptides have primarily been developed on the basis of molecular dynamics (MD) simulation.^16−18^ Most MD research involves identifying conformations in aqueous solutions or organic solvents. Furthermore, there are complex studies simulating the membrane permeation process of cyclic peptides across a lipid bilayer.^19,20^ Cyclic peptides tend to exist in various conformations, resulting in slow conformational transitions relative to simulation time scales.^18^ As a result, many accelerated sampling techniques have been used, such as replica exchange MD (REMD).^21^ In addition, analyzing simulation results by methods such as Markov state models (MSMs) can provide information on the behavior of cyclic peptides that are important for elucidating the mechanism of membrane permeation and structural optimization to increase membrane permeability.^22^ However, the computational cost of the MD simulation method is still high.

In contrast to previously used methods, machine learning-based prediction methods can provide rapid predictions. However, previous reports using machine learning prediction methods did not yield a reliable level of generalization performance due to insufficient training data.^23,24^ For instance, Digiesi et al. used 62 cyclic hexapeptides that have very similar structures to each other.^23^ The topological polar surface area (TPSA) of these peptides ranged from approximately 150 to 250 Å^2^, which could only cover a very narrow chemical space. The lack of available databases that collect structurally diverse cyclic peptides is a major reason for poor generalization performance and is currently the greatest obstacle to developing comprehensive machine learning predictions. The combinations of amino acids that comprise cyclic peptides are numerous; therefore, the chemical space of possible cyclic peptides is very large. Furthermore, even a single residue change in the amino acid sequence can lead to drastic changes in membrane permeability.^25^ Most studies using the available data fixed the majority of structures and measured changes in membrane permeability with very few residue changes.^22,26^ Therefore, building a machine learning prediction model with high generalization performance requires a large amount of structurally diverse training data collected from a larger body of publications than employed in previous studies.

In this study, we constructed CycPeptMPDB, a comprehensive membrane permeability database for cyclic peptides with the aim of developing a machine learning-based prediction model and a computational method for cyclic peptide drug design. Because several previous publications reported various measurement experiments for the permeability data, information such as the measurement experiment system was recorded. We generated basic 2D SMILES representations and 3D structures (not crystal structures but computationally generated conformations) of cyclic peptides. Furthermore, to unambiguously represent cyclic peptides, we organized the sequence information from each source and unified the name of the monomer (substructure, such as the residue). Then, we used the hierarchical editing language for macromolecules (HELM) notation to generate sequence representations for all peptides.

## Methods

### Overview of CycPeptMPDB Framework

**Figure 1:**
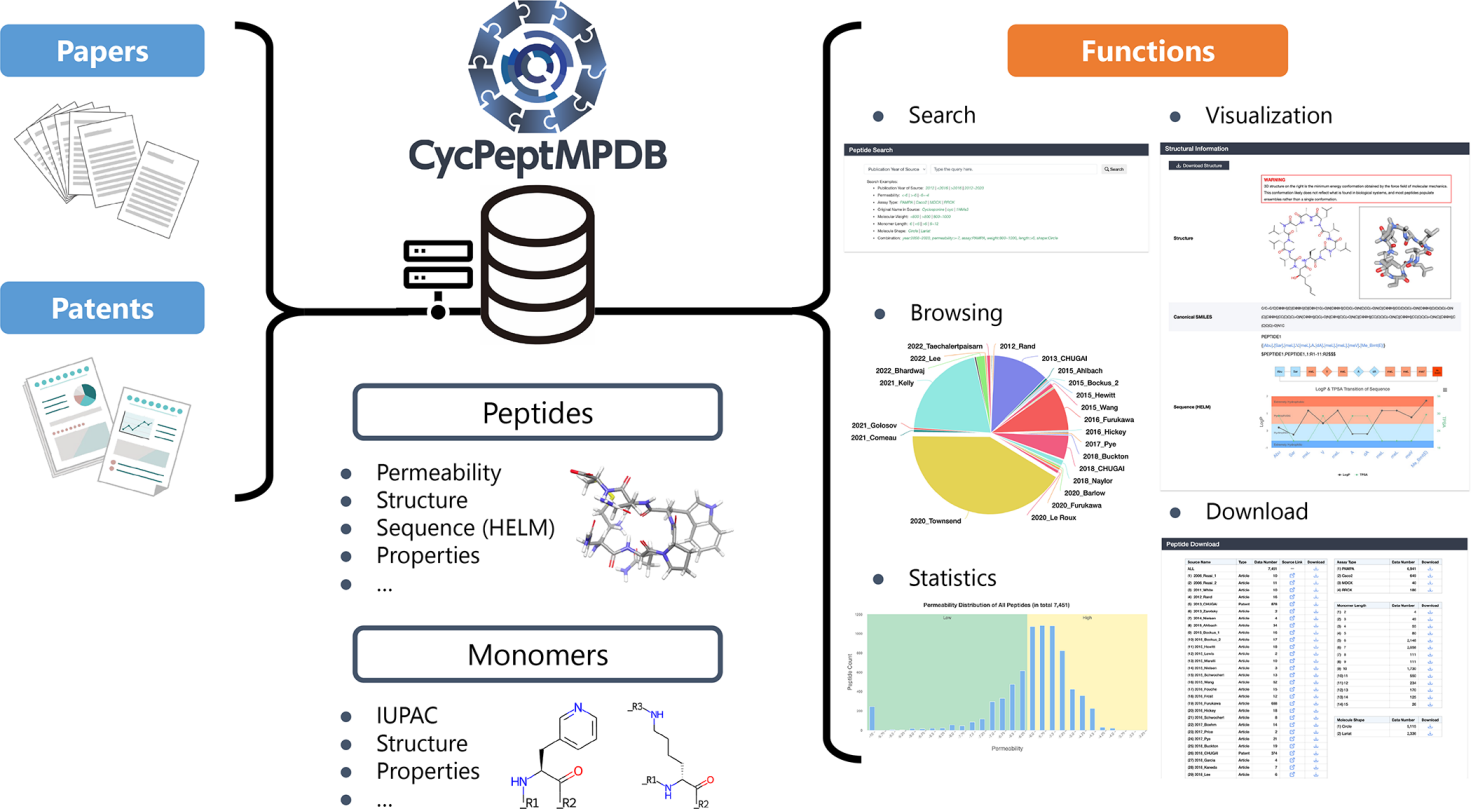
Basic framework of CycPeptMPDB. CycPeptMPDB data were collected from published papers and patents of pharmaceutical companies and then manually inspected. Information in various formats was deposited into a PostgreSQL-based database for visualization, downloading, and other web-based functions.

As shown in Figure 1, CycPeptMPDB is a comprehensive database recording the membrane permeability of cyclic peptides based on data obtained from published papers and pharmaceutical patents. It mainly contains two types of data for cyclic peptides: (1) property information, i.e., experimental values of membrane permeability and physical quantities such as LogP (an index of lipophilicity) estimated from chemical structure, and (2) chemical structure information, i.e., sequence information described by HELM and monomers as partial structures constituting the cyclic peptides. CycPeptMPDB provides several functions such as data storage, statistics and visualization, searching and analysis, and downloading.

### Data Collection

We collected a total of 7334 structurally diverse cyclic peptide data points (the number of peptides including duplicated structures from all publication sources was 7451) and their measured membrane permeability from 45 published papers and 2 patents from pharmaceutical companies. The source list of CycPeptMPDB is shown in the Table S1. Membrane permeability in CycPeptMPDB was expressed as a log-scaled value LogP_exp_. For peptides whose membrane permeability could not be measured due to the detection limit, etc., LogP_exp_ was set to the minimum value, −10.0 (1.0 × 10^–10^ cm/s, detailed records such as the detection limit can be viewed on the peptide detail page). The structures of peptides were recorded in CycPeptMPDB using the SMILES notation. Structural errors in the original publication were corrected (for example, when the SMILES structure attached to the publication differs from the sequence described in the publication, the structure was corrected based on the sequence information). When there was a new source directly citing the membrane permeability values of the old source, the number of these data was counted only in the old source. As shown in Table S1, most previously reported studies included a relatively small number of peptides, with only six publications reporting on over 100 peptides. In addition, as mentioned above, most of the publications deal with structurally similar cyclic peptides; therefore, the molecular weight range of peptides reported in a single paper is relatively narrow. By collecting more than 40 publications, we were able to build a database of cyclic peptides covering a wide range of molecular weights (from 342.4 to 1777.7) and TPSA (from 73.0 to 702.0 Å^2^). Molecular weight and TPSA were the MolWt and TPSA descriptor calculated by RDKit software, respectively. In the selected publications, there were 6941 measurements by PAMPA, 649 measurements by Caco-2 assay, 40 measurements by Madin-Darby canine kidney (MDCK) assay, and 186 measurements by Ralph Russ canine kidney (RRCK) assay. All measured values were recorded when measurements obtained by multiple assays were reported in a single publication. Of all identified peptides, the membrane permeability measurements of 365 peptides were determined using two different assays. When a peptide was measured by two assays in a single publication, the permeability of the assay with more data was used as the representative membrane permeability measurement. If the permeabilities from both assays were similar, the representative value was determined according to the following assay rank: (1) PAMPA, (2) Caco-2, (3) RRCK, and (4) MDCK. Furthermore, a detailed description of the assay protocol used in each study was also recorded as experimental conditions such as reaction time and initial concentration affect membrane permeability measurements.

**Figure 2:**
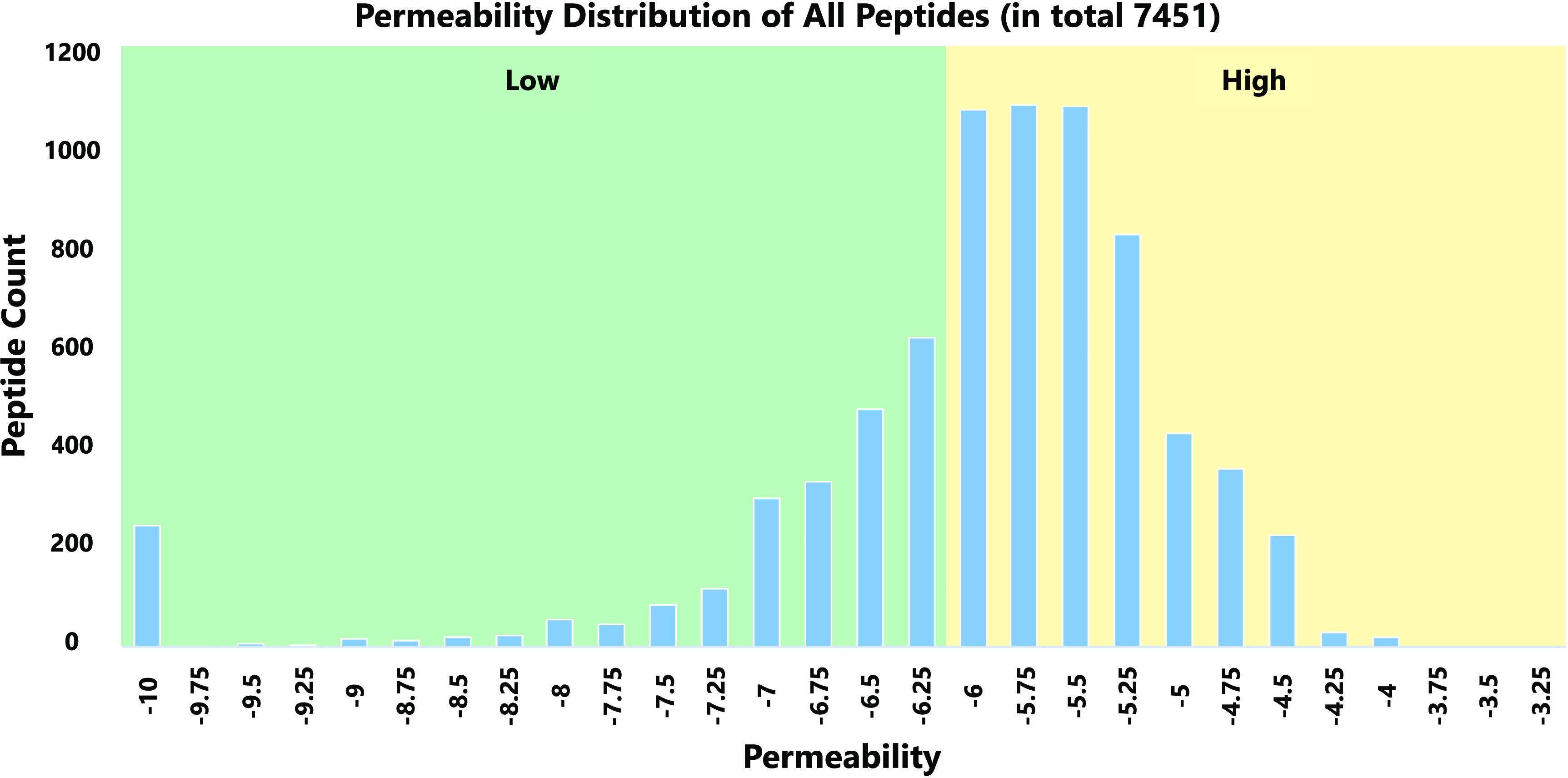
Permeability (LogP_exp_) distribution of all peptides. The background color of the high permeability range is yellow, and the background color of the low permeability range is green.

Moreover, to improve the readability of the list display, collected cyclic peptides were classified into two types by membrane permeability (Figure 2). Cyclic peptides with LogP_exp_ higher than or equal to −6.00 (1.0 × 10^–6^cm/s) are generally considered to have good permeability and were classified as high (5113 peptides). Conversely, cyclic peptides with LogP_exp_ below −6.00 were classified as low (2338 peptides).

### 3D Structure Generation of Cyclic Peptides

Chemical structural information on the collected cyclic peptides was recorded in SMILES notation. Additionally, as conformation generation for cyclic peptides is computationally expensive, we generated the 3D structure of each cyclic peptide using RDKit software (version 2020.09.1) allowing users to quickly start relevant research. We generated 5000 conformations per peptide and removed redundant conformations with RMSD less than 1.0 Å. Next, the structure optimization of each conformation was performed using the UFF force field, and the top structure with the lowest potential energy was selected. This approach provided a computationally efficient way to obtain the 3D structure of cyclic peptides. However, it should be noted that the minimum energy conformation obtained by molecular mechanics force fields may not necessarily reflect the true conformations of the peptides in biological systems. Furthermore, most peptides are likely to populate conformational ensembles rather than a single conformation. The 3D structure of the cyclic peptide can be viewed online and downloaded in SDF format.

### Sequence Representation of Cyclic Peptides by HELM

**Figure 3:**
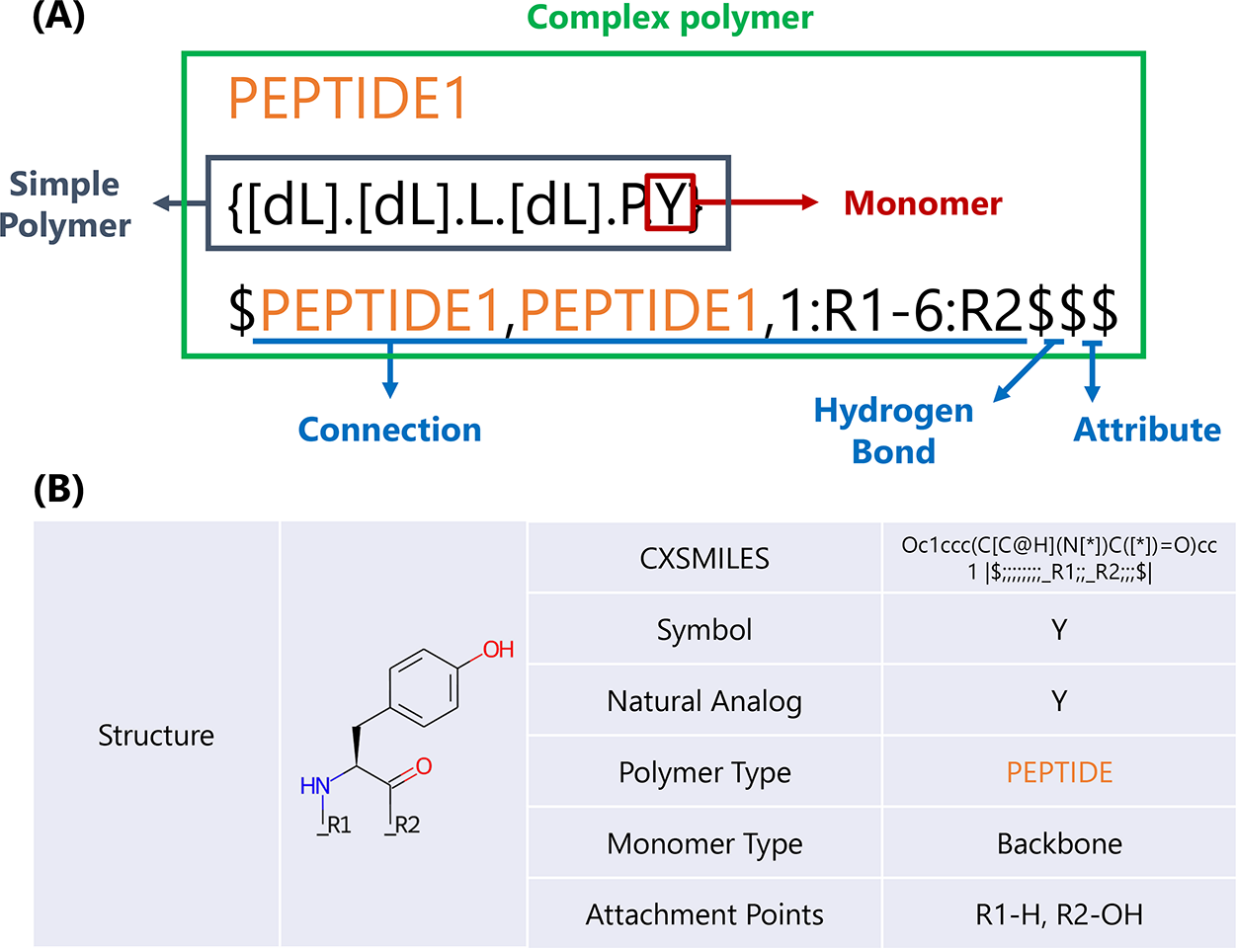
(A) Example of HELM notation (PEPTIDE1[dL].[dL].L.[dL].P.Y$PEPTIDE1, PEPTIDE1,1:R1-6:R2$$$) and its constituent parts in this study. If the simple polymer is a peptide, write the simple polymer as PEPTIDEx (where x is a number, and in the case of RNA is RNAx). The connection section means that R1 of the first monomer of PEPTIDE1 and R2 of the sixth monomer of PEPTIDE1 are connected. The hydrogen bonds and attributes sections of all peptides in this study are empty. (B) Example of monomer definition of tyrosine (Y).

Cyclic peptides are relatively large compared to small molecules, and appropriate sequence representation is essential for good readability. Therefore, we used the HELM notation to generate a unified sequence representation of collected cyclic peptides. HELM can hierarchically represent complex structures with relatively high molecular weights, such as antisense oligonucleotides, short interference RNAs, peptides, proteins, and antibody drug conjugates.^27^ HELM consists of four level hierarchies: complex polymer, simple polymer, monomer, and atom (Figure 3(A)). First, a complex polymer expresses information about the chemical structure of the entire macromolecule. Its components are simple polymers and their connections (including hydrogen bonds and attributes). Second, a simple polymer is composed of monomers of the same polymer type. A simple polymer is defined as a single linear chain; branching and cycling structures are not covered in this hierarchy. Certain polymer types have explicit rules for connections between monomers, and the position and rules of connections can express the direction of monomer sequences (e.g., PEPTIDE notation represents amino acid sequences from N-terminus to C-terminus). Moreover, monomers are composed of atoms and bonds and can be represented in formats such as Molfile and CXSMILES (Figure 3(B), Chemaxon Extended SMILES). Each monomer was given a unique symbol similar to the amino acid code represented in the peptide sequences (e.g., A, G). Here, the definition of monomer also includes the positions of its connections (i.e., attachment points). When describing linear peptides, the original HELM definition dictates that monomers are connected by peptide bonds. The attachment point R1 is defined as the N atom of the amino group, and R2 is defined as the C atom of the carboxyl group (the attachment points after R3 is the branch of the side chain). R1 and R2 in the terminal can only be used to form the main chain of the linear peptide (R1 and R3, R2 and R3 can form a ring). Contrary to the original definition, the N-terminus and C-terminus of linear peptides are often connected in the case of cyclic peptides. Therefore, in this study, N-terminal R1 and C-terminal R2 were able to be used to form a ring (like 1:R1–6:R2 in Figure 3(A), HELM in PubChem and ChEMBL databases is also like our definition). In addition, the O atom changed from the N atom was also set as R1 because there were many cyclic peptides with amide-to-ester substitutions.

### Monomer Definition in CycPeptMPDB

**Figure 4:**
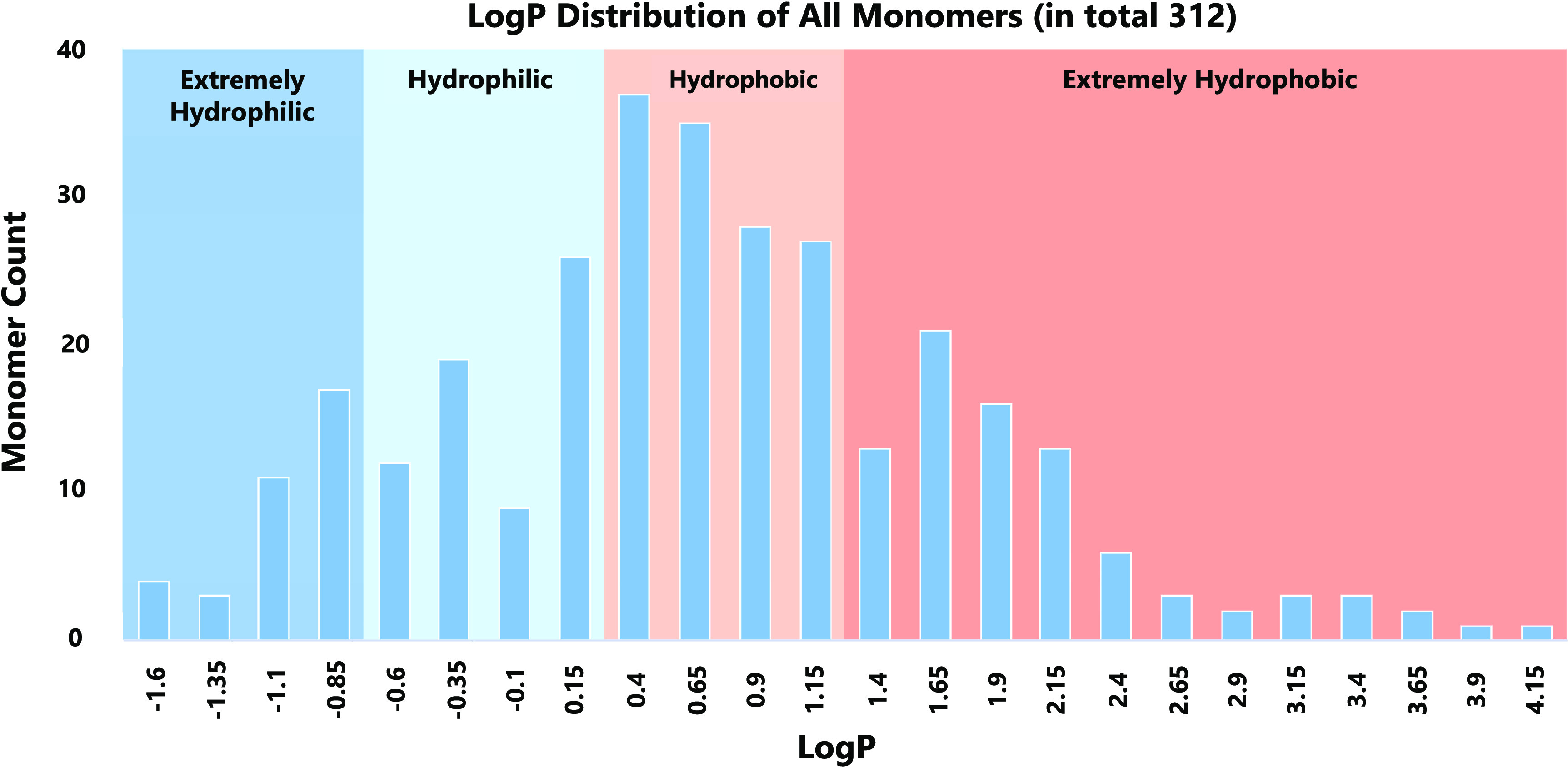
LogP distribution of all monomers. The background color for extremely hydrophilic monomers is blue, hydrophilic monomers light blue, hydrophobic monomers orange, and extremely hydrophobic monomers red.

As mentioned in the previous section, the method used to define monomers that comprise peptides is important and should be standardized for all entries in the database. However, many of the selected publications did not record sequence representations, and even when records were available, the representation of special amino acids tended to differ notably between these publications. Therefore, we defined the partial structure obtained after cleaving the peptide bonds and ester bonds of the cyclic peptide as a monomer (Figure 3(B), CycPeptMPDB has no peptide containing disulfide bonds). As a result, a total of 312 types of monomers were obtained. There were 305 monomers with the backbone monomer type (having two or more attachment points) and 7 monomers with the terminal monomer type (used for terminal modification of peptide sequences with only one attachment point). Monomers were described in CXSMILES to represent positions of attachment points. For monomers included in the PubChem database, their general compound and International Union of Pure and Applied Chemistry (IUPAC) names were recorded. When not included in the PubChem database, their IUPAC names were generated from SMILES using STOUT software (version 2.0, https://github.com/Kohulan/Smiles-TO-iUpac-Translator).^28^ Furthermore, when setting the symbol (the monomers short display name in HELM) and natural analog of monomers, we referred the PubChem database and the monomer library of ChEMBL database (version 29, contained 2851 types of monomers). At this stage, there were 112 types of monomers that did not have suitable symbols, and their symbols were set as Mono1 to Mono112. The explanation of the naming method of symbol is shown in the Table 1. Additionally, we defined two types of peptide molecule shapes: Circle and Lariat. This classification was based on HELM sequence information. Peptides with cyclization positions at both the N- and C-terminal ends of the sequence were considered Circle peptides, and peptides with cyclization positions not at the end of the sequence, i.e., cyclized between a terminal and a side chain, were considered Lariat peptides. Moreover, when calculating descriptors for monomers, the presence of hydrogen bond donors and acceptors for the amide and carboxyl groups may not accurately represent the partial physicochemical properties of the cyclic peptide before cleavage. Therefore, if the original attachment point atom was N or O (amide-to-ester substitution), it was capped with methyl (CH3). If the original attachment point atom was C, a hydrogen atom (H) was added. All monomer descriptors shown in CycPeptMPDB were calculated from such processed molecules. Similar to the method of classifying cyclic peptides by membrane permeability described in the Data Collection section, monomers were classified into four types by LogP (Wildman–Crippen atom-based LogP value, MolLogP descriptor calculated by RDKit software) based on the LogP values of natural amino acids (Figure 4). Monomers with LogP < −0.60 were set as extremely hydrophilic (35 monomers, lower than G: −0.60). Those with −0.60 ≤ LogP < 0.40 were set as hydrophilic (66 monomers, lower than V: 0.43, general hydrophilic amino acids, such as G: −0.60, A: −0.21, and P: 0.28). Those with 0.40 ≤ LogP < 1.40 were set as hydrophobic (127 monomers, general hydrophobic amino acids, such as V: 0.43, I: 0.82, L: 0.82, and F: 1.02). Those with 1.40 ≤ LogP were set as extremely hydrophobic (84 monomers, extremely hydrophobic amino acids, such as W: 1.50).

**Table 1:** Explanation of Symbols Naming Method

Explanation of naming method	Example of symbol
1. Natural amino acids	A, L, dV
2. Monomers with a general compound name	Abu, Sar, dCha
3. Monomers with side chain modifications	Ala(tBu), dGlu(OMe), dPhe(4-F)
4. Monomers with N-terminal modifications	Me_Cha, Bn_Gly, 3-pyridylethyl_Gly
5. Monomers with C-terminal modifications	Glu_NH2
6. Monomers with amide-to-ester substitution	(N->O)Val, d(N->O)Val
7. Combination of above 1 to 7	Me_Phe(3-Cl), Cys(EtO2H)_NH2
8. Terminal modification of cyclic peptides	ac-, -pip
9. Monomers could not named by 1 to 8	Mono1 – Mono112

## Results and Discussion

### Peptide Browsing Function

**Figure 5:**
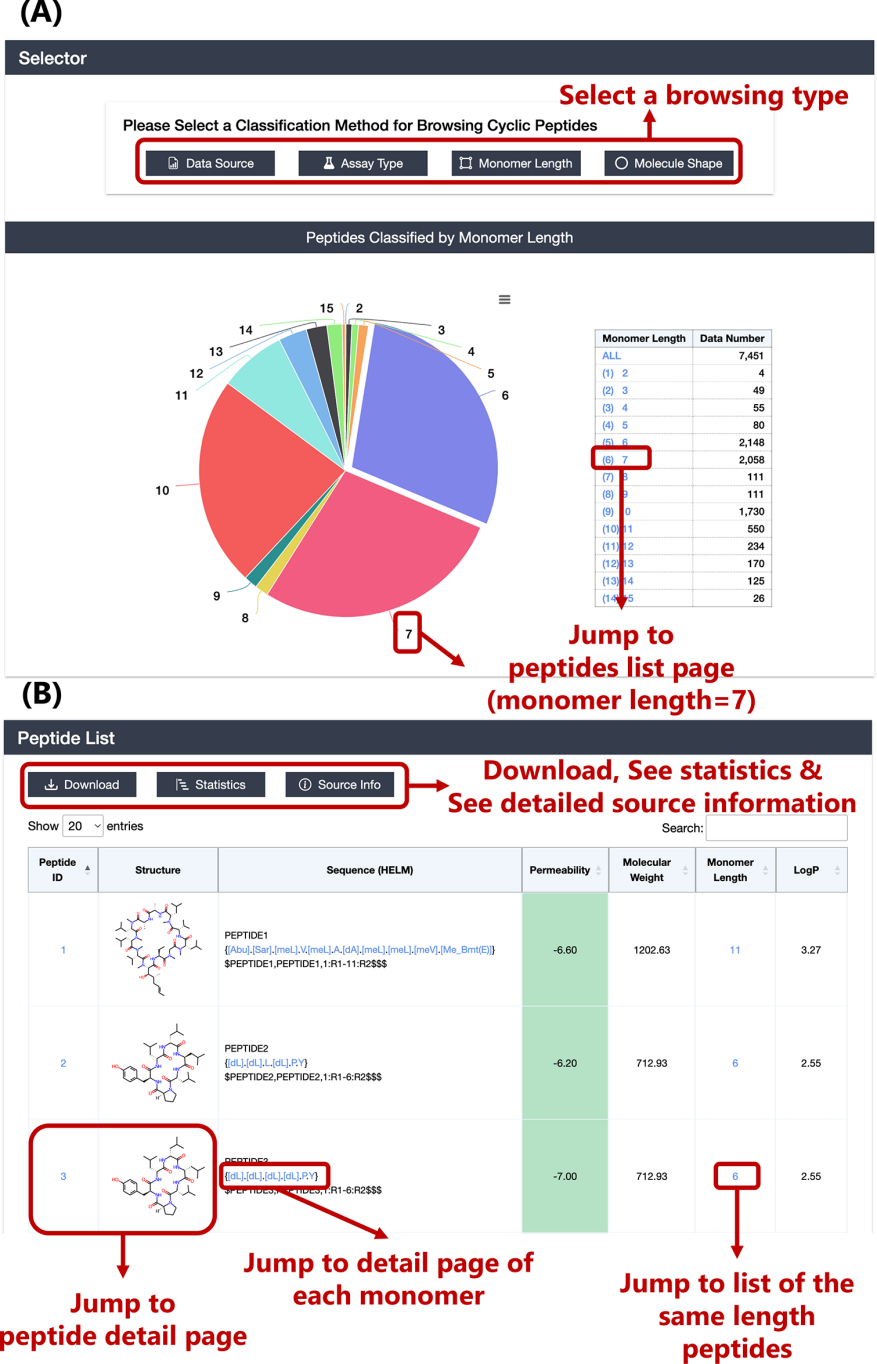
(A) Classification method selection for browsing peptides and browsing page. The case when monomer length is selected is shown as an example. (B) Peptides list page. The background color of the permeability cell is yellow when the permeability is high (LogP_exp_ ≥ −6.00) and green when it is low (LogP_exp_ < −6.00).

As mentioned previously, CycPeptMPDB includes 7334 structurally diverse cyclic peptides (the number including duplicated structures from all publications was 7451) from 45 papers and 2 pharmaceutical company patents. As shown in Table S1, only six publications reported more than 100 peptides, 2020_Townsend^29^ with 3086 peptides accounting for more than 40% of the total. In addition, when browsing peptides, we prepared three classification methods in addition to browsing by data source: assay type, monomer length, and molecule shape (Figure 5(A)). When classified by monomer length (peptide sequence length), according to the monomer splitting method used in this study (cleavage of peptide and ester bond), the monomer length ranged from 2 to 15. Furthermore, the behavior of side chains of cyclic peptides, and the formation of hydrogen bonds between side chains and the main chain, can have a significant impact on membrane permeability; therefore, it may be necessary to separate the treatment of Circle and Lariat peptides. Circle peptides accounted for under 70% (5115) of the total and Lariat peptides for about 30% (2336). Moreover, detailed information for the source can also be accessed if browsed by data source, as shown in Figure 5(B). After navigating to the corresponding subset list page, the brief table of peptides displays basic information on peptides, including CycPeptMPDB ID, 2D structure image, HELM, permeability, molecular weight, monomer length, and LogP (Figure 5(B)). If users want to further refine the list of accessed peptides, the search function on the upper right of the table can be used. This search function differs from the search function described in the Peptide Search Function for Quick Data Retrieval section in that it can filter peptides that partially match the contents of the table (data source name, publication year of source, original name in source, and molecule shape are provided in addition to the table contents).

### Peptide Search Function for Quick Data Retrieval

In addition to peptide browsing, users can use the peptide search function to quickly find their target peptides. The search module supports conditional searches for peptides by seven options and their logical combinations. These options include publication year of source, permeability, assay type, original compound name in source, molecular weight, monomer length, and molecule shape. Numeric options such as permeability can also be searched by range, see CycPeptMPDB usage for specific search examples and detailed instructions. In addition to the homepage, users can also use the peptide search function from the search box in the upper right corner of each page.

### Visualization Functions on Peptide Detail Page

**Figure 6:**
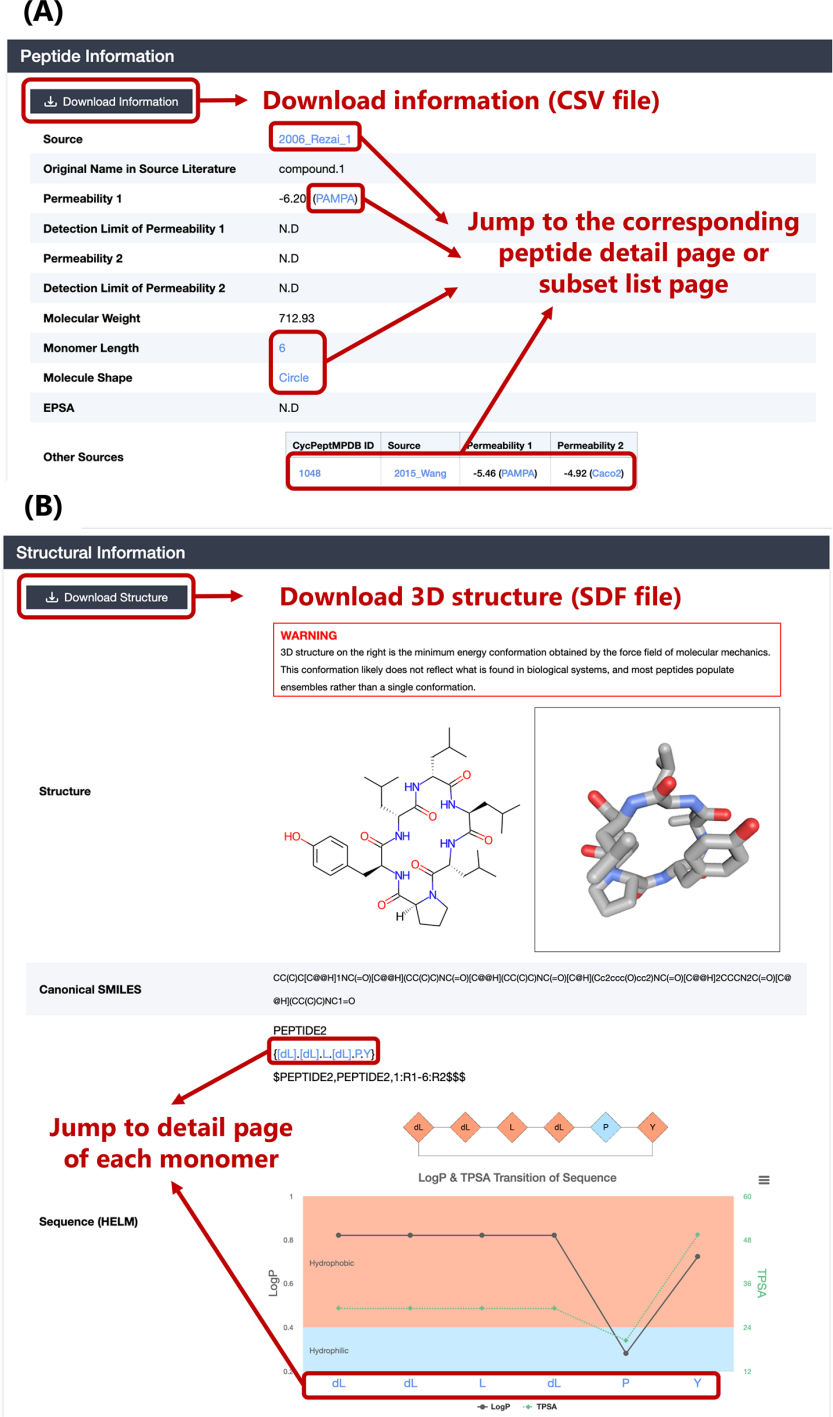
(A) Peptide information section of peptide detail page. (B) Structural information section of peptide detail page. HELM images and LogP transition diagrams are colored by the LogP value of each monomer.

We incorporated several useful functions in the peptide detail page for peptides and monomers. First, for peptides of the same structure reported in multiple sources, we listed all published membrane permeability measurements in the peptide information section (Figure 6(A)). In addition to measurements from different assays of the same source, the measured membrane permeabilities between each source are also different. This function allows users to quickly select the measured membrane permeability values obtained under different measurement environments. Because the number of 3D structures that cyclic peptides can take is enormous, the generation of 3D structures requires a large amount of computational resources. Therefore, to facilitate the use of CycPeptMPDB, we generated 5000 conformations per peptide with RDKit software as described in the 3D Structure Generation of Cyclic Peptides section. The most stable single conformation was selected and stored (Figure 6(B)). Finally, to increase the readability of the HELM representation and support sequence-based analysis, we also created HELM image and LogP and TPSA transition diagrams for the sequence (Figure 6(B)). By using these functions, users can quickly capture the change in peptide sequence and partial characterization.

### Browsing and Visualization Functions of Monomers

**Figure 7:**
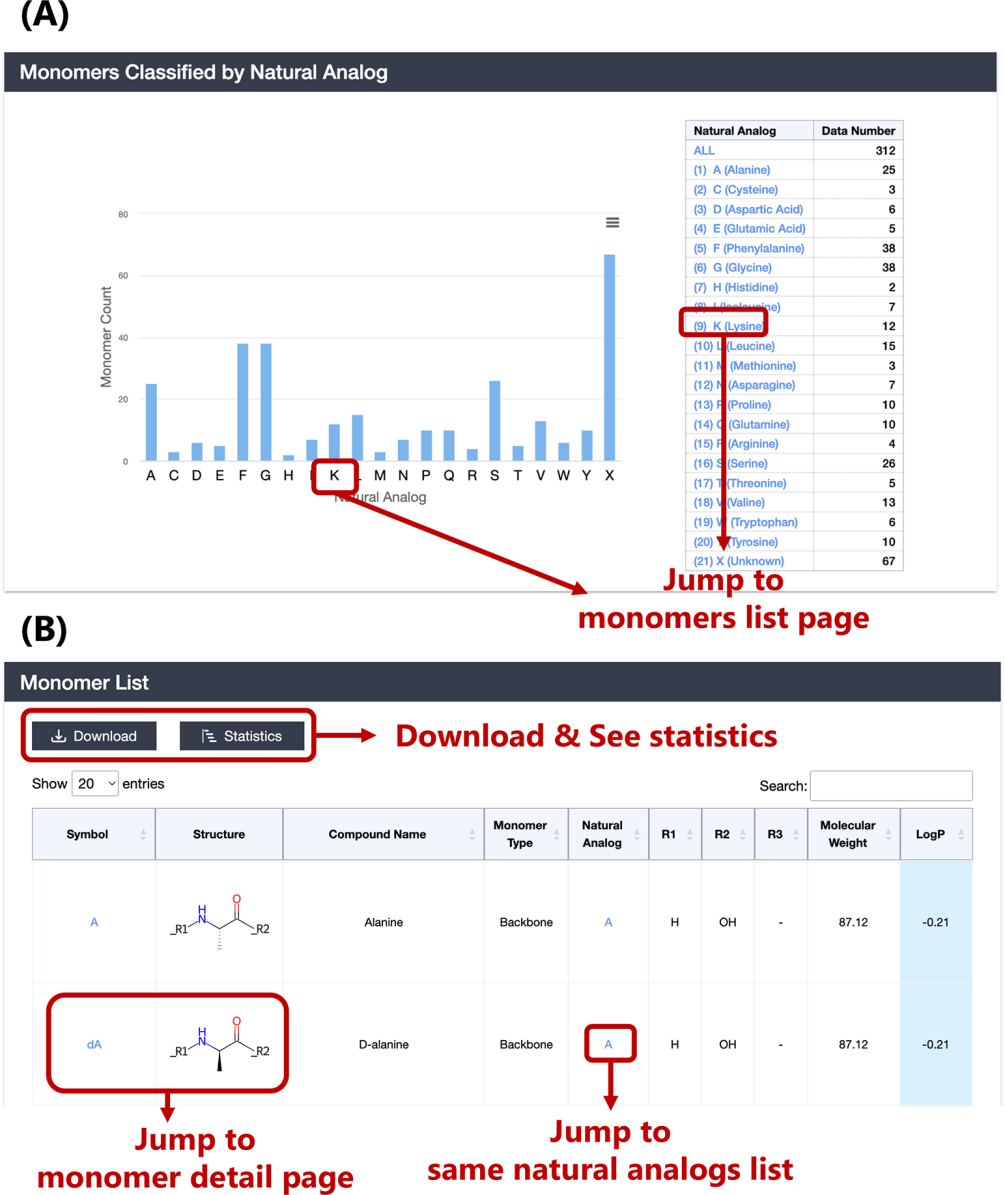
Monomer (A) browsing and (B) list pages. LogP cell background color is blue when LogP is extremely hydrophilic (LogP < −0.60), light blue when hydrophilic (−0.60 ≤ LogP < 0.40), orange when hydrophobic (0.40 ≤ LogP < 1.40), and red when extremely hydrophobic (1.40 ≤ LogP).

**Figure 8:**
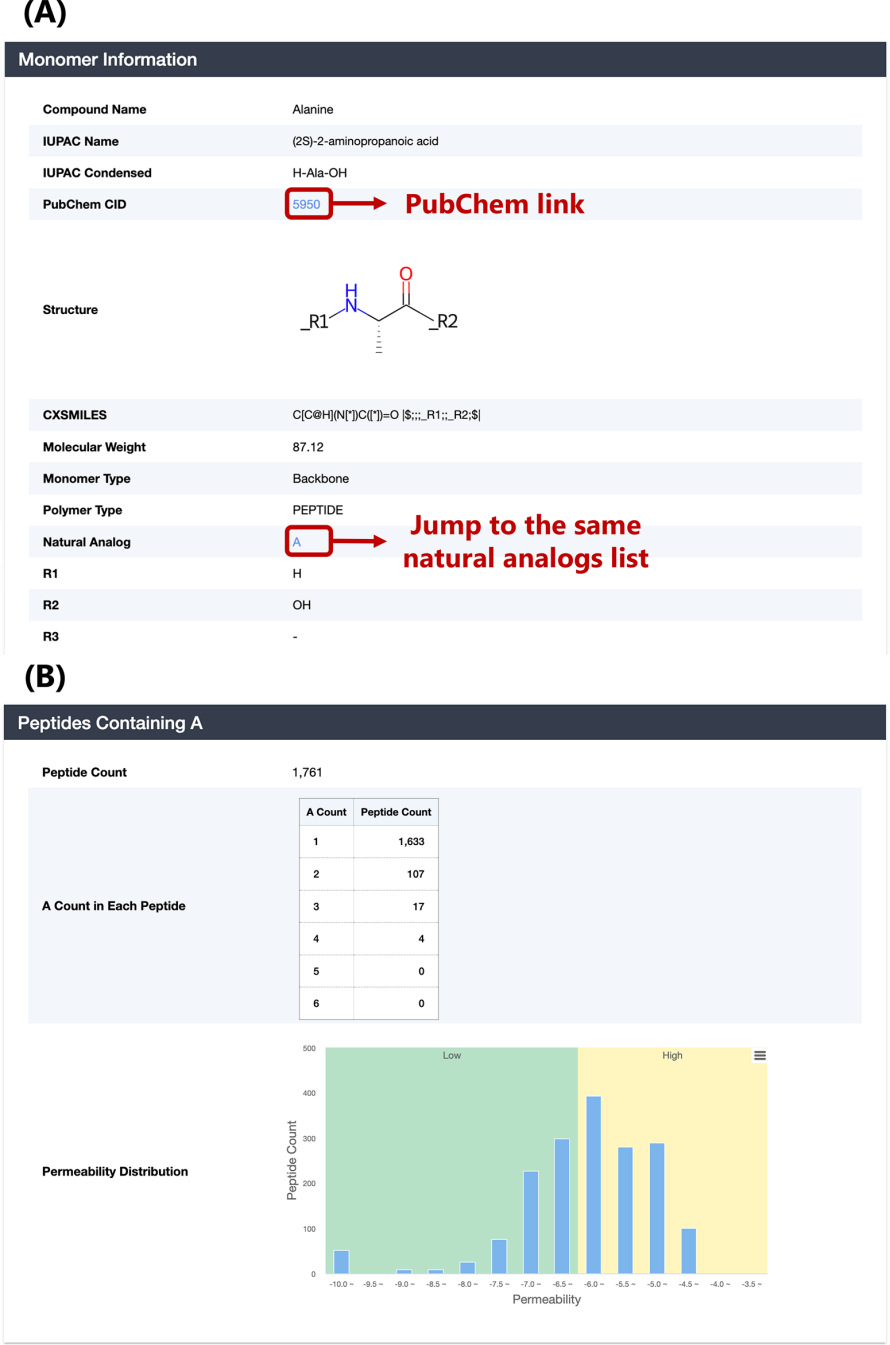
(A) Monomer information section of monomer detail page. (B) Statistics section of peptides containing current monomer.

A total of 312 monomers were defined as substructures that comprise the peptides, and they were classified into 21 categories by their natural analog (20 natural amino acids and unknown (X)). Natural analogs were established by referring to the description of each monomer in PubChem and the monomer library of ChEMBL. Among these 21 categories, categories F (38) and G (38) included the most monomers, and there were other two categories with more than 20 monomers: A (25) and S (26). We provided a browsing function for monomers by natural analog (Figure 7(A)). After navigating to the corresponding subset list page, the brief table displays basic information on monomers, including symbol, 2D structure image, monomer type (backbone or terminal), natural analog, attachment points (R1–R3), molecular weight, and LogP (Figure 7(B)). Next, as shown in Figure 8(A), we included the PubChem CID of the monomer and created a link to PubChem in the monomer detail page. Users can obtain more diverse information on the monomer from PubChem. Moreover, the monomer detail page lists the distribution of the number of peptides containing each monomer and the membrane permeability distribution of these peptides (Figure 8(B)). This function will assist users in performing monomer-level analysis.

## Conclusion

This study reports our development of CycPeptMPDB, a comprehensive database of membrane permeability measurements of cyclic peptides with a web interface, consisting of 7334 cyclic peptides collected from 47 publications. In addition to providing a peptide’s component information, sequence representations essential for cyclic peptide drug discovery were created using HELM notation, and monomers that comprise cyclic peptides were constructed. Browsing and searching functions are incorporated to facilitate the rapid acquisition of targeted peptides. CycPeptMPDB provides several additional functions such as online data visualization, data analysis, and downloading, making it a useful platform to support membrane permeability studies. We will continue to collect membrane permeability data of cyclic peptides and record them in CycPeptMPDB. Future improvements to the CycPeptMPDB online analysis platform will include an improved user-friendly interface and more integrative functions. We collected over 7000 structurally diverse cyclic peptide data and have enabled the development of a machine learning-based membrane permeability prediction model with reliable generalization performance. Next, we intend to develop a prediction model that accounts for mechanisms specific to cyclic peptide membrane permeation, such as the “chameleonic” property. At that time, it may be necessary to generate conformers not only from a single environment but from both water and membrane-mimicking environments (such as chloroform).

## Data Availability

All information recorded in CycPeptMPDB can be downloaded from http://cycpeptmpdb.com/download/. The structure and membrane permeability of all cyclic peptides recorded in CycPeptMPDB were collected from published papers and patents. The list of source publications is shown in the Supporting Information (Table S1) or http://cycpeptmpdb.com/resources/statistics/. The implementations of CycPeptMPDB used Docker (https://www.docker.com/). All data were stored in a PostgreSQL-based database and managed by pgAdmin4 (version 6.14, https://www.pgadmin.org/). The website was implemented by Django (version 3.2, https://www.djangoproject.com/), a high-level web framework with Python (version 3.8.3). The web page was constructed using HTML, CSS, and JavaScript; dynamic chart visualization was performed using Highcharts (https://www.highcharts.com/), and 3D structures were presented using ChemDoodle Web Components (https://web.chemdoodle.com/). In addition, RDKit software (version 2020.09.1, https://www.rdkit.org/) was used for 3D structure generation of cyclic peptides, descriptor calculation of cyclic peptides and monomers, and 2D structures image generation of cyclic peptides and monomers. Furthermore, as mentioned in the Methods section, the IUPAC names of monomers referred to the PubChem database (https://pubchem.ncbi.nlm.nih.gov/) were included, and some of them were generated by STOUT software (version 2.0, https://github.com/Kohulan/Smiles-TO-iUpac-Translator).^28^

## Supporting Information Available

Source list for CycPeptMPDB (Table S1): number of peptides, molecular weight range, and assay type of membrane permeability for each source (PDF)
