# Supplementary material for: CycPeptMPDB: A Comprehensive Database of Membrane Permeability of Cyclic Peptides
Source: J Chem Inf Model. 2023 Mar 17;63(7):2240–50. doi: 10.1021/acs.jcim.2c01573 (PMC10091415; doi:10.1021/acs.jcim.2c01573)
Supplement: Supplementary file 1 — ci2c01573_si_001.pdf [file ci2c01573_si_001.pdf]

# Supporting Information

## CycPeptMPDB: A Comprehensive Database of Membrane Permeability of Cyclic Peptides

Jianan Li[1], Keisuke Yanagisawa[1, 2], Masatake Sugita[1, 2], Takuya Fujie[1, 2], Masahito Ohue[1, 2], and Yutaka Akiyama[1, 2, *]

1. Department of Computer Science, School of Computing, Tokyo Institute of Technology, Meguro-ku, Tokyo 152-8550, Japan
2. Middle-Molecule IT-based Drug Discovery Laboratory (MIDL), Tokyo Institute of Technology, Meguro-ku, Tokyo 152-8550, Japan

* To whom correspondence should be addressed.
Contact: akiyama@c.titech.ac.jp

# 1 Supplemental Tables

**Table S1**: Source literature list for CycPeptMPDB. The number of peptides, molecular weight range, and assay type of membrane permeability for each source are shown.

| Source | Peptides | Molecular weight | PAMPA | Caco-2 | RRCK | MDCK |
|---|---|---|---|---|---|---|
| 2006_Rezai_1 [1] | 10 | 712.9 – 1202.6 | ✓ | | | |
| 2006_Rezai_2 [2] | 11 | 710.9 – 840.1 | ✓ | | | |
| 2011_White [3] | 10 | 712.9 – 1202.6 | | | ✓ | |
| 2012_Rand [4] | 16 | 712.9 – 828.1 | | ✓ | ✓ | |
| 2013_CHUGAI [5] | 878 | 813.0 – 1777.7 | ✓ | | | |
| 2013_Zaretsky [6] | 2 | 471.6 – 627.8 | | ✓ | | |
| 2014_Nielsen [7] | 4 | 709.9 – 778.0 | | | ✓ | |
| 2015_Ahlbach [8] | 34 | 414.5 – 1620.7 | ✓ | | | |
| 2015_Bockus_1 [9] | 16 | 755.0 – 1202.6 | ✓ | | ✓ | |
| 2015_Bockus_2 [10] | 17 | 707.9 – 778.0 | ✓ | ✓ | | |
| 2015_Hewitt [11] | 18 | 712.9 – 755.0 | | ✓ | | |
| 2015_Lewis [12] | 2 | 712.9 – 755.0 | ✓ | | | ✓ |
| 2015_Marelli [13] | 10 | 454.5 – 849.0 | ✓ | ✓ | | |
| 2015_Nielsen [14] | 3 | 724.9 – 785.1 | ✓ | | | |
| 2015_Schwochert [15] | 13 | 712.9 – 793.0 | | | ✓ | |
| 2015_Wang [16] | 62 | 454.5 – 882.1 | ✓ | ✓ | | |
| 2016_Fouché [17] | 15 | 790.9 – 1199.6 | | | | ✓ |
| 2016_Frost [18] | 12 | 542.6 – 699.9 | ✓ | | | |
| 2016_Furukawa [19] | 688 | 606.8 – 944.2 | ✓ | ✓ | | |
| 2016_Hickey [20] | 18 | 662.8 – 1202.6 | ✓ | | | |
| 2016_Schwochert [21] | 8 | 696.9 – 731.0 | | | | ✓ |
| 2017_Boehm [22] | 14 | 848.1 – 929.1 | | | ✓ | |
| 2017_Price [23] | 2 | 1019.4 – 1202.6 | | | ✓ | |
| 2017_Pye [24] | 21 | 785.0 – 1151.5 | ✓ | | ✓ | |
| 2018_Buckton [25] | 19 | 596.7 – 744.8 | ✓ | ✓ | | |
| 2018_CHUGAI [26] | 374 | 1062.3 – 1664.2 | | ✓ | | |
| 2018_García-Pindado [27] | 4 | 725.9 – 883.7 | ✓ | | | |
| 2018_Kaneda [28] | 7 | 842.1 – 870.1 | ✓ | | | |
| 2018_Lee [29] | 6 | 639.7 – 653.7 | ✓ | | | |
| 2018_Naylor [30] | 81 | 578.8 – 1218.6 | ✓ | | ✓ | |
| 2018_Ramalho [31] | 10 | 767.0 – 851.0 | ✓ | ✓ | | |
| 2019_Ono [32] | 8 | 712.9 – 712.9 | ✓ | | | |
| 2020_Barlow [33] | 26 | 537.7 – 753.0 | ✓ | | | |
| 2020_Furukawa [34] | 36 | 987.3 – 1197.5 | ✓ | | | ✓ |
| 2020_Hosono [35] | 11 | 712.9 – 727.0 | ✓ | | | |
| 2020_Le Roux [36] | 47 | 342.4 – 486.7 | ✓ | ✓ | | |
| 2020_Townsend [37] | 3086 | 654.9 – 958.2 | ✓ | | | |
| 2021_Comeau [38] | 42 | 430.5 – 458.6 | ✓ | ✓ | | |
| 2021_Golosov [39] | 27 | 758.0 – 1076.5 | ✓ | | ✓ | |
| 2021_Kelly [40] | 1519 | 974.3 – 1220.4 | ✓ | | | ✓ |
| 2021_Lee [41] | 5 | 1160.6 – 1231.7 | ✓ | ✓ | | |
| 2021_Wang [42] | 24 | 959.2 – 1169.5 | ✓ | | | |
| 2022_Bhardwaj [43] | 136 | 622.8 – 1299.7 | ✓ | ✓ | | |
| 2022_Lee [44] | 24 | 1160.6 – 1251.7 | ✓ | | | |
| 2022_Saunders [45] | 11 | 542.6 – 623.7 | ✓ | | | |
| 2022_Taechalertpaisarn [46] | 52 | 661.8 – 856.1 | ✓ | | | |
| 2022_Tamura [47] | 12 | 792.0 – 950.2 | ✓ | | | |

# References


[1] Rezai, T., Yu, B., Millhauser, G. L., Jacobson, M. P., and Lokey, R. S. (2006a). Testing the conformational hypothesis of passive membrane permeability using synthetic cyclic peptide diastereomers. *J. Am. Chem. Soc.,* **128**(8), 2510–2511.

[2] Rezai, T., Bock, J. E., Zhou, M. V., Kalyanaraman, C., Lokey, R. S., and Jacobson, M. P. (2006b). Conformational flexibility, internal hydrogen bonding, and passive membrane permeability: Successful in silico prediction of the relative permeabilities of cyclic peptides. *J. Am. Chem. Soc.,* **128**(43), 14073–14080.

[3] White, T. R., Renzelman, C. M., Rand, A. C., Rezai, T., McEwen, C. M., Gelev, V. M., Turner, R. A., Linington, R. G., Leung, S. S., Kalgutkar, A. S., Bauman, J. N., Zhang, Y., Liras, S., Price, D. A., Mathiowetz, A. M., Jacobson, M. P., and Lokey, R. S. (2011). On-resin N-methylation of cyclic peptides for discovery of orally bioavailable scaffolds. *Nat. Chem. Biol.,* **7**(11), 810–817.

[4] Rand, A. C., Leung, S. S. F., Eng, H., Rotter, C. J., Sharma, R., Kalgutkar, A. S., Zhang, Y., Varma, M. V., Farley, K. A., Khunte, B., Limberakis, C., Price, D. A., Liras, S., Mathiowetz, A. M., Jacobson, M. P., and Lokey, R. S. (2012). Optimizing PK properties of cyclic peptides: the effect of side chain substitutions on permeability and clearance. *MedChemComm*, **3**(10), 1282–1289.

[5] Chugai Pharma. Co., Ltd. (2013). Peptide compound cyclization method. *Patent WO2013100132A1.*

[6] Zaretsky, S., Scully, C. C., Lough, A. J., and Yudin, A. K. (2013). Exocyclic control of turn induction in macrocyclic peptide scaffolds. *Chem. Eur. J.,* **19**(52), 17668–17672.

[7] Nielsen, D. S., Hoang, H. N., Lohman, R. J., Hill, T. A., Lucke, A. J., Craik, D. J., Edmonds, D. J., Griffith, D. A., Rotter, C. J., Ruggeri, R. B., Price, D. A., Liras, S., and Fairlie, D. P. (2014). Improving on Nature: Making a Cyclic Heptapeptide Orally Bioavailable. Angew. *Chem. Int. Ed.,* **53**(45), 12059–12063.

[8] Ahlbach, C. L., Lexa, K. W., Bockus, A. T., Chen, V., Crews, P., Jacobson, M. P., and Lokey, R. S. (2015). Beyond cyclosporine A: conformation-dependent passive membrane permeabilities of cyclic peptide natural products. *Future Med. Chem.,* **7**(16), 2121–2130.

[9] Bockus, A. T., Lexa, K. W., Pye, C. R., Kalgutkar, A. S., Gardner, J. W., Hund, K. C., Hewitt, W. M., Schwochert, J. A., Glassey, E., Price, D. A., Mathiowetz, A. M., Liras, S., Jacobson, M. P., and Lokey, R. S. (2015a). Probing the Physicochemical Boundaries of Cell Permeability and Oral Bioavailability in Lipophilic Macrocycles Inspired by Natural Products. J. *Med. Chem.,* **58**(11), 4581–4589.

[10] Bockus, A. T., Schwochert, J. A., Pye, C. R., Townsend, C. E., Sok, V., Bednarek, M. A.,

and Lokey, R. S. (2015b). Going Out on a Limb: Delineating the Effects of β-Branching, N-Methylation, and Side Chain Size on the Passive Permeability, Solubility, and Flexibility of Sanguinamide A Analogues. *J. Med. Chem.,* **58**(18), 7409–7418.

[11] Hewitt, W. M., Leung, S. S., Pye, C. R., Ponkey, A. R., Bednarek, M., Jacobson, M. P., and Lokey, R. S. (2015). Cell-permeable cyclic peptides from synthetic libraries inspired by natural products. *J. Am. Chem. Soc.,* **137**(2), 715–721.

[12] Lewis, I., Schaefer, M., Wagner, T., Oberer, L., Sager, E., Wipfli, P., and Vorherr, T. (2015). A detailed investigation on conformation, permeability and PK properties of two related cyclohexapeptides. *Int. J. Pept. Res. Ther.,* **21**(2), 205–221.

[13] Marelli, U. K., Bezençon, J., Puig, E., Ernst, B., and Kessler, H. (2015). Enantiomeric cyclic peptides with different caco-2 permeability suggest carrier-mediated transport. *Chem. Eur. J.,* **21**(22), 8023–8027.

[14] Nielsen, D. S., Lohman, R. J., Hoang, H. N., Hill, T. A., Jones, A., Lucke, A. J., and Fairlie, D. P. (2015). Flexibility versus Rigidity for Orally Bioavailable Cyclic Hexapeptides. *ChemBioChem*, **16**(16), 2289–2293.

[15] Schwochert, J., Turner, R., Thang, M., Berkeley, R. F., Ponkey, A. R., Rodriguez, K. M., Leung, S. S., Khunte, B., Goetz, G., Limberakis, C., Kalgutkar, A. S., Eng, H., Shapiro, M. J., Mathiowetz, A. M., Price, D. A., Liras, S., Jacobson, M. P., and Lokey, R. S. (2015). Peptide to Peptoid Substitutions Increase Cell Permeability in Cyclic Hexapeptides. *Org. Lett.,* **17**(12), 2928–2931.

[16] Wang, C. K., Northfield, S. E., Swedberg, J. E., Colless, B., Chaousis, S., Price, D. A., Liras, S., and Craik, D. J. (2015). Exploring experimental and computational markers of cyclic peptides: Charting islands of permeability. *Eur. J. Med. Chem.,* **97**, 202–213.

[17] Fouché, M., Schäfer, M., Berghausen, J., Desrayaud, S., Blatter, M., Piéchon, P., Dix, I., Martingarcia, A., and Roth, H. J. (2016). Design and Development of a Cyclic Decapeptide Scaffold with Suitable Properties for Bioavailability and Oral Exposure. *ChemMedChem*, **11**(10), 1048–1059.

[18] Frost, J. R., Scully, C. C., and Yudin, A. K. (2016). Oxadiazole grafts in peptide macrocycles. *Nat. Chem.,* **8**(12), 1105–1111.

[19] Furukawa, A., Townsend, C. E., Schwochert, J., Pye, C. R., Bednarek, M. A., and Lokey, R. S. (2016). Passive Membrane Permeability in Cyclic Peptomer Scaffolds Is Robust to Extensive Variation in Side Chain Functionality and Backbone Geometry. *J. Med. Chem.,* **59**(20), 9503–9512.

[20] Hickey, J. L., Zaretsky, S., St Denis, M. A., Kumar Chakka, S., Morshed, M. M., Scully, C. C., Roughton, A. L., and Yudin, A. K. (2016). Passive Membrane Permeability of Macrocycles Can Be Controlled by Exocyclic Amide Bonds. *J. Med. Chem.,* **59**(11), 5368–

5376.

[21] Schwochert, J., Lao, Y., Pye, C. R., Naylor, M. R., Desai, P. V., Gonzalez Valcarcel, I. C., Barrett, J. A., Sawada, G., Blanco, M. J., and Lokey, R. S. (2016). Stereochemistry Balances Cell Permeability and Solubility in the Naturally Derived Phepropeptin Cyclic Peptides. *ACS Med. Chem. Lett.,* **7**(8), 757–761.

[22] Boehm, M., Beaumont, K., Jones, R., Kalgutkar, A. S., Zhang, L., Atkinson, K., Bai, G., Brown, J. A., Eng, H., Goetz, G. H., Holder, B. R., Khunte, B., Lazzaro, S., Limberakis, C., Ryu, S., Shapiro, M. J., Tylaska, L., Yan, J., Turner, R., Leung, S. S., Ramaseshan, M., Price, D. A., Liras, S., Jacobson, M. P., Earp, D. J., Lokey, R. S., Mathiowetz, A. M., and Menhaji-Klotz, E. (2017). Discovery of Potent and Orally Bioavailable Macrocyclic Peptide-Peptoid Hybrid CXCR7 Modulators. *J. Med. Chem.,* **60**(23), 9653−9663.

[23] Price, D. A., Eng, H., Farley, K. A., Goetz, G. H., Huang, Y., Jiao, Z., Kalgutkar, A. S., Kablaoui, N. M., Khunte, B., Liras, S., Limberakis, C., Mathiowetz, A. M., Ruggeri, R. B., Quan, J. M., and Yang, Z. (2017). Comparative pharmacokinetic profile of cyclosporine (CsA) with a decapeptide and a linear analogue. *Org. Biomol. Chem.,* **15**(12), 2501–2506.

[24] Pye, C. R., Hewitt, W. M., Schwochert, J., Haddad, T. D., Townsend, C. E., Etienne, L., Lao, Y., Limberakis, C., Furukawa, A., Mathiowetz, A. M., Price, D. A., Liras, S., and Lokey, R. S. (2017). Nonclassical Size Dependence of Permeation Defines Bounds for Passive Adsorption of Large Drug Molecules. *J. Med. Chem.,* **60**(5), 1665–1672.

[25] Buckton, L. K. and McAlpine, S. R. (2018). Improving the Cell Permeability of Polar Cyclic Peptides by Replacing Residues with Alkylated Amino Acids, Asparagines, and d-Amino Acids. *Org. Lett.,* **20**(3), 506–509.

[26] Chugai Pharma. Co., Ltd. (2018). Cyclic peptide compound having high membrane permeability, and library containing same. *Patent WO2018225864A1.*

[27] García-Pindado, J., Willemse, T., Goss, R., Maes, B. U., Giralt, E., Ballet, S., and Teixidó, M. (2018). Bromotryptophans and their incorporation in cyclic and bicyclic privileged peptides. *Biopolymers,* **109**(10), e23112.

[28] Kaneda, M., Kawaguchi, S., Fujii, N., Ohno, H., and Oishi, S. (2018). Structure-Activity Relationship Study on Odoamide: Insights into the Bioactivities of Aurilide-Family Hybrid Peptide-Polyketides. *ACS Med. Chem. Lett.,* **9**(4), 365–369.

[29] Lee, L. L., Buckton, L. K., and McAlpine, S. R. (2018). Converting polar cyclic peptides into membrane permeable molecules using N-methylation. *Pept. Sci.,* **110**(3), e24063.

[30] Naylor, M. R., Ly, A. M., Handford, M. J., Ramos, D. P., Pye, C. R., Furukawa, A., Klein, V. G., Noland, R. P., Edmondson, Q., Turmon, A. C., Hewitt, W. M., Schwochert, J., Townsend, C. E., Kelly, C. N., Blanco, M. J., and Lokey, R. S. (2018). Lipophilic Permeability Efficiency Reconciles the Opposing Roles of Lipophilicity in Membrane

Permeability and Aqueous Solubility. *J. Med. Chem.,* **61**(24), 11169–11182.

[31] Ramalho, S. D., Wang, C. K., King, G. J., Byriel, K. A., Huang, Y. H., Bolzani, V. S., and Craik, D. J. (2018). Synthesis, Racemic X-ray Crystallographic, and Permeability Studies of Bioactive Orbitides from Jatropha Species. *J. Nat. Prod.,* **81**(11), 2436–2445.

[32] Ono, S., Naylor, M. R., Townsend, C. E., Okumura, C., Okada, O., and Lokey, R. S. (2019). Conformation and Permeability: Cyclic Hexapeptide Diastereomers. *J. Chem. Inf. Model.,* **59**(6), 2952–2963.

[33] Barlow, N., Chalmers, D. K., Williams-Noonan, B. J., Thompson, P. E., Norton, R. S., and Thompson, P. E. (2020). Improving Membrane Permeation in the beyond Rule-of-Five Space by Using Prodrugs to Mask Hydrogen Bond Donors. *ACS Chem. Biol.,* **15**(8), 2070–2078.

[34] Furukawa, A., Schwochert, J., Pye, C. R., Asano, D., Edmondson, Q. D., Turmon, A. C., Klein, V. G., Ono, S., Okada, O., and Lokey, R. S. (2020). Drug-Like Properties in Macrocycles above MW 1000: Backbone Rigidity versus Side-Chain Lipophilicity. *Angew. Chem. Int. Ed.,* **132**(48), 21755–21761.

[35] Hosono, Y., Morimoto, J., Townsend, C. E., Kelly, C. N., Naylor, M. R., Lee, H. W., Scott Lokey, R., and Sando, S. (2020). Amide-to-Ester Substitution Improves Membrane Permeability of a Cyclic Peptide without Altering Its Three-Dimensional Structure. *ChemRxiv*. DOI: 10.26434/chemrxiv.12272861.v1.

[36] Le Roux, A., Blaise, É., Boudreault, P. L., Comeau, C., Doucet, A., Giarrusso, M., Collin, M. P., Neubauer, T., Kölling, F., Göller, A. H., Seep, L., Tshitenge, D. T., Wittwer, M., Kullmann, M., Hillisch, A., Mittendorf, J., and Marsault, E. (2020). Structure-Permeability Relationship of Semipeptidic Macrocycles-Understanding and Optimizing Passive Permeability and Efflux Ratio. *J. Med. Chem.,* **63**(13), 6774–6783.

[37] Townsend, C. E., Naylor, M. R., Jason, E., Pye, C. R., Schwochert, J. A., Edmondson, Q., and Lokey, R. S. (2020). The passive permeability landscape around geometrically diverse hexa- And heptapeptide macrocycles. *ChemRxiv*, pages 1–21. DOI: 10.26434/chemrxiv.13335941.v1.

[38] Comeau, C., Ries, B., Stadelmann, T., Tremblay, J., Poulet, S., Fröhlich, U., Côté, J., Boudreault, P. L., Derbali, R. M., Sarret, P., Grandbois, M., Leclair, G., Riniker, S., and Marsault, É. (2021). Modulation of the Passive Permeability of Semipeptidic Macrocycles: N- And C-Methylations Fine-Tune Conformation and Properties. *J. Med. Chem.,* **64**(9), 5365–5383.

[39] Golosov, A. A., Flyer, A. N., Amin, J., Babu, C., Gampe, C., Li, J., Liu, E., Nakajima, K., Nettleton, D., Patel, T. J., Reid, P. C., Yang, L., and Monovich, L. G. (2021). Design of Thioether Cyclic Peptide Scaffolds with Passive Permeability and Oral Exposure. *J. Med.*

*Chem.,* **64**(5), 2622–2633.

[40] Kelly, C. N., Townsend, C. E., Jain, A. N., Naylor, M. R., Pye, C. R., Schwochert, J., and Lokey, R. S. (2021). Geometrically Diverse Lariat Peptide Scaffolds Reveal an Untapped Chemical Space of High Membrane Permeability. *J. Am. Chem. Soc.,* **143**(2), 705–714.

[41] Lee, D., Lee, S., Choi, J., Song, Y. K., Kim, M. J., Shin, D. S., Bae, M. A., Kim, Y. C., Park, C. J., Lee, K. R., Choi, J. H., and Seo, J. (2021). Interplay among Conformation, Intramolecular Hydrogen Bonds, and Chameleonicity in the Membrane Permeability and Cyclophilin A Binding of Macrocyclic Peptide Cyclosporin O Derivatives. *J. Med. Chem.*, **64**(12), 8272-8286.

[42] Wang, S., König, G., Roth, H.-J., Fouché, M., Rodde, S., and Riniker, S. (2021). Effect of Flexibility, Lipophilicity, and the Location of Polar Residues on the Passive Membrane Permeability of a Series of Cyclic Decapeptides. *J. Med. Chem.,* **64**(17), 12761–12773.

[43] Bhardwaj, G., O 'Connor, J., Rettie, S., Huang, Y. -H., Ramelot, T. A., Mulligan, V. K., Alpkilic, G. G., Palmer, J., Bera, A. K., Bick, M. J., Di Piazza, M., Li, X., Hosseinzadeh, P., Craven, T. W., Tejero, R., Lauko, A., Choi, R., Glynn, C., Dong, L., Griffin, R., van Voorhis, W. C., Rodriguez, J., Stewart, L., Montelione, G. T., Craik, D., and Baker, D. (2022). Accurate de novo design of membrane-traversing macrocycles. *Cell*, **185**(19), 3520–3532.

[44] Lee, D., Kang, J. A., Lim, C., Bae, S., Choi, J., Park, M., Kim, Y. C., Cho, Y., Park, S. G., and Seo, J. (2022). Entry inhibition of hepatitis B virus using cyclosporin O derivatives with peptoid side chain incorporation. *Bioorg. Med. Chem.,* 116862.

[45] Saunders, G. J. and Yudin, A. K. (2022). Property-driven development of passively permeable macrocyclic scaffolds using heterocycles. *Angew. Chem. Int. Ed.,* **134**(33), e202206866.

[46] Taechalertpaisarn, J., Ono, S., Okada, O., Johnstone, T. C., and Lokey, R. S. (2022). A New Amino Acid for Improving Permeability and Solubility in Macrocyclic Peptides through Side Chain-to-Backbone Hydrogen Bonding. *J. Med. Chem.,* **65**(6), 5072–5084.

[47] Tamura, T., Inoue, M., Yoshimitsu, Y., Hashimoto, I., Ohashi, N., Tsumura, K., Suzuki, K., Watanabe, T., and Hohsaka, T. (2022). Chemical synthesis and cell-free expression of thiazoline ring-bridged cyclic peptides and their properties on biomembrane permeability. *Bull. Chem. Soc. Jpn.,* **95**(2), 359–366.
